# Female-mediated selective sperm activation may remodel major histocompatibility complex-based mate choice decisions in humans

**DOI:** 10.1038/s41437-025-00759-9

**Published:** 2025-05-09

**Authors:** Annalaura Jokiniemi, Tanja Turunen, Mikko Kohonen, Martina Magris, Jarmo Ritari, Liisa Kuusipalo, Jukka Partanen, Jukka Kekäläinen

**Affiliations:** 1 Department of Environmental and Biological Sciences, University of Eastern Finland, Joensuu, Finland; 2 Finnish Red Cross Blood Service, Research and Development, Helsinki, Finland; 3 North Karelia Central Hospital, Joensuu, Finland

**Keywords:** Sexual selection, Evolutionary genetics, Evolutionary biology

## Abstract

Major histocompatibility complex (MHC) genes are known to mediate mate choice both at the individual and gamete level. However, it has remained unclear how different episodes of MHC-associated mate choice interact and contribute to the total selection on MHC genes. Here, we clarified this interaction in humans by performing a full-factorial experiment where 10 females first ranked the attractiveness and intensity of the body odours of 11 males. Then we studied whether female odour preferences in these same 110 male-female combinations predicted sperm performance in the presence of follicular fluid (sperm-stimulating female reproductive fluid). When analyzing the total MHC similarity (including classical and non-classical MHC genes) of the male-female combinations, we found that females preferred the body odours of MHC-similar males, but that sperm motility was positively affected by the MHC dissimilarity of the male-female combinations. No associations were found for classical MHC genes only. Furthermore, odour preferences were negatively associated with sperm motility at the end of the follicular fluid treatment. Together, our results indicate that individual and gamete-level mate choice processes may act in opposing directions and that the most attractive males are not necessarily the most optimal partners at the post-copulatory level. Finally, our findings suggest that gamete-mediated mate choice may have a definitive role in disfavouring genetically incompatible partners from fertilizing oocytes.

## Introduction

Finding a mating partner is a key requirement for sexually reproducing organisms. In many species, mate choice is based on chemical signals, and olfactory cues play an especially important role in the process (Restrepo et al. 2004; Fedina and Lewis 2007; Kekäläinen et al. 2011; Amo et al. 2012). Accordingly, earlier studies have demonstrated that olfactory cues are sexually dimorphic and allow individuals to discriminate their potential mating partners even in species that are otherwise sexually monomorphic (e.g. Penn and Potts 1998; Ferkin 2018). Supporting this view, olfactory cues have been shown to convey detailed information of the various characteristics of the prospective mates, such as sex, age, dominance status, familiarity, and the health status (Penn and Potts 1998; Charlton 2013; Kavaliers and Choleris 2017). It has also been demonstrated that individuals have their own distinctive body odour (Penn et al. 2007), which can reveal information about genetic similarity (Roberts et al. 2005a).

Several studies have demonstrated that the attractiveness of the body odour is strongly affected by the genes of the major histocompatibility complex (MHC, termed human leucocyte antigen, HLA, in humans) and that males and females typically mate non-randomly with respect to their MHC genotype (Penn and Potts, 1998; Penn 2002; Milinski et al. 2005). Olfactory-based sensing of MHC genotype has been demonstrated to occur via peptide antigens presented by MHC molecules (Milinski et al. 2013), while other studies have suggested that MHC molecules may affect odour indirectly by binding volatile compounds or by shaping host’s microbiota composition (Grogan et al. 2019). Given that sufficient MHC diversity is important for the development of offspring immunocompetence, MHC-based mate choice is often thought to favour MHC-dissimilar partners. However, early theoretical models (e.g. Nowak et al. 1992) and later experimental studies (e.g. Aeschlimann et al. 2003; Wegner et al. 2003) have demonstrated that individuals that possess intermediate (optimal) number of different MHC molecules have the highest probability of mounting immune response against prevailing pathogens.

Supporting the immunological benefits of male-female MHC-dissimilarity, olfactory-based mating preferences for MHC-dissimilar mating partners have been demonstrated to occur in a number of species, including mice (Penn and Potts 1999), lizards (Olsson et al. 2003), bats (Santos et al. 2016), birds (Grieves et al. 2019) and non-human primates (Grogan et al. 2019). However, in line with the MHC optimality hypothesis, olfactory cues have also been demonstrated to mediate mate choice towards intermediate MHC dissimilarity (fish: Reusch et al. 2001; Aeschlimann et al. 2003, see also Forsberg et al. 2007) and against both of the above-mentioned hypotheses, other studies have found odour-based mating preferences towards MHC-similar partners (bird: Leclaire et al. 2017; badger: Sin et al. 2015, see also Slade et al. 2019). Similarly, in humans, many studies have shown that women prefer the body odours of MHC dissimilar males (Wedekind et al. 1995; Wedekind and Furi 1997; Roberts et al. 2008; Sorokowska et al. 2018), whereas other studies have found evidence for both MHC-optimality and MHC-similarity preferences (Jacob et al. 2002; Winternitz et al. 2017; Milinski 2022). Together, these findings imply that MHC-associated mate choice may be context-dependent and/or sensitive to the life history stage, such as age of the study subjects (e.g. Havlicek and Roberts 2009).

In many species, mate choice continues after the copulation in the form of cryptic female choice, where females use various physical or chemical mechanisms to bias paternity towards the sperm of particular males (Fitzpatrick and Lüpold 2014; Firman et al. 2017; Lymbery et al. 2017; Gasparini et al. 2020; Hadlow et al. 2020). Along with the above-mentioned pre-mating mate choice, MHC genes have been demonstrated to play an important role also in cryptic female choice (e.g. Rülicke et al. 1998; Yeates et al. 2009; Løvlie et al. 2013; Gessner et al. 2017). Earlier studies have found that cryptic female choice can bias fertilisation towards MHC-dissimilar males in red junglefowl (Løvlie et al. 2013), whereas in fishes, it has been demonstrated to favour MHC-similar males (Atlantic salmon: Yeates et al. 2009; Guppies: Gasparini et al. 2015; Chinook salmon: Gessner et al. 2017) or intermediate MHC-diversity (three-spined stickleback: Lenz et al. 2018). MHC-based cryptic female choice is known to be mediated by various female reproductive fluids that facilitate mate choice at the level of the gametes (gamete-mediated mate choice, GMMC: reviewed by Kekäläinen and Evans 2018). GMMC potentially enables much more accurate mate choice than any pre-mating sexual selection mechanisms (Kekäläinen and Evans 2018), and can operate even in the absence of MHC-dependent (pre-mating) mate choice (Lenz et al. 2018). Furthermore, given that GMMC occurs later than any pre-mating mate choice process, it can have a major role in determining the overall strength and direction of sexual selection.

Despite the widespread evidence of the importance of cryptic female choice (and GMMC) in a number of animal taxa (e.g. Gasparini and Pilastro 2011; Evans et al. 2012; Rosengrave et al. 2016), it has been unclear whether cryptic female choice could occur in humans. Interestingly, Fitzpatrick et al. (2020) recently demonstrated in humans that sperm attraction (chemotaxis) towards the follicular fluid of different females was dependent on the male-female combination, and that follicular fluid selectively attracted sperm from specific males over others. Furthermore, Jokiniemi et al. (2020a, 2020b) showed (also in humans) that sperm physiological response towards female reproductive tract secretions (follicular fluid and cervical mucus) is stronger in HLA-dissimilar male-female combinations than in more similar combinations. Together, these findings indicate that the female reproductive tract may mediate cryptic female choice towards the sperm of genetically compatible males (Kekäläinen 2021; Magris et al. 2021). Supporting this conclusion, it has been demonstrated that sperm physiological response (speed enhancement and chemotaxis) to follicular fluid predicts the fertilisation success of the sperm (Ralt et al. 1991) and that follicular fluid facilitate sperm capability to fertilise the oocyte (e.g. Hong et al. 1993). Furthermore, it has been shown that sperm motility and swimming velocity are strong predictors of both natural and assisted conception in humans (e.g. Tomlinson et al. 2013). Therefore, follicular fluid-induced changes in sperm motility traits can be expected to act as key indicators of cryptic female choice in humans (when experimental fertilisation of the oocytes is not ethically possible).

Numerous studies have investigated the mechanisms of pre-mating or post-mating sexual selection separately, but much less is known about how these sexual selection processes interact and contribute to overall reproductive success (Evans and Garcia-Gonzalez 2016). Consequently, to the best of our knowledge, only Mahdjoub et al. (2023) have attempted to investigate the combined effect of pre- and post-mating sexual selection. Authors showed in *Drosophila melanogaster* that both male overall reproductive value (‘quality’) and male-female compatibility influenced mating and reproductive success, but that their relative importance varied between pre- vs. post-mating episodes of sexual selection. Furthermore, Fitzpatrick et al. (2020) recently tested whether males’ sperm preferentially swim (chemotaxis) towards the follicular fluids of their partners and found no differences in sperm responsiveness between partners and non-partners. On the other hand, the genetic basis of their results remained unclear, and authors also did not experimentally test actual pre-mating preferences.

Here, we investigated whether female pre-mating preferences for male body odours predict their preferences at the level of the gametes. We conducted a full-factorial (North Carolina II) experimental design between 11 males and 10 females (110 male-female combinations). All the females rated the attractiveness of male body odour samples during their fertile days. We then measured sperm performance after follicular fluid treatment (i.e. after combining sperm aliquots with follicular fluid samples from different females) in these same male-female combinations and tested whether pre-mating odour preferences predicted sperm performance in the presence of follicular fluid. We also genotyped all the participants by a genome-wide single-nucleotide polymorphism (SNP) array and imputed their HLA class I and II alleles. Finally, we investigated whether male-female HLA similarity or genetic relatedness (kinship) predicted the above-mentioned pre- and post-copulatory mating preferences.

## Material and methods

### Ethical approval of the study

The collection of all biological samples (odour, semen, follicular fluid, and blood) and all the procedures of this study were approved by the Research Ethics Committee of the Hospital District of Northern Savo, Finland (77/2017). All participants volunteered and signed an informed consent before enroling in the study. All of them received compensation (120€ and 80€, for males and females, respectively) for participation in the study. Males received higher compensation than females because of the arduousness of the odour collection procedure and associated everyday restrictions (see below).

### Participants of the study and male odour collection

Male participants (*n* = 11) were recruited via the infertility clinic of North Karelia Central Hospital (Joensuu, Finland) and the University of Eastern Finland. The mean age of participating males was 33.2 ( ± 4.33 s.d., range 26–39) years. Males collected odour samples in September-October 2020 and donated sperm samples in March–April 2021. All the males had a normal semen quality according to World Health Organisation’s criteria for cell count (concentration >15 million/ml), motility (motile cells > 40%), and viability (live cells > 58%) (World Health Organization, 2021). Males received written instructions for odour collection, along with all the material needed for the collection of their odour and sperm samples (Supplementary Methods 1). Three days before the initiation of the odour collection procedure, male participants ceased using any antiperspirants and only used odourless deodorant instead. During the last 24 h prior to the odour collection, participants ceased to use the deodorant, used only odourless hygiene products and avoided eating aromatic food (Supplementary Methods 1). All the males had unshaven armpits, and all of them reported that they had been following given instructions.

Just before the odour collection period, males washed their bodies and hair with odourless shower gel and shampoo (LV®, Berner Oy). Axillary odour samples were then collected with 100% cotton pads (size 7 × 9 cm), which were taped to both armpits for 24 h (one pad per armpit, Supplementary Fig. 1). To avoid potential external odour contamination of the pads, males were also asked to use cotton t-shirts (pre-washed with odourless detergent, LV®) during the whole odour collection period. Furthermore, all the participants were asked to avoid exercising and intimate contact with their partners. After the odour collection, pads from both armpits were separately wrapped in foil, placed in zip bags, and stored at −80 °C. Given that human odour samples can be stored up to several months without significant changes in odour pleasantness (Lenochova et al. 2009), it is likely that the storage time of collected odour samples did not bias female odour ratings (see below).

The female participants (*n* = 10) were recruited from the infertility clinic of North Karelia Central Hospital. All of them had regular menstrual cycles, were non-smokers, and were not using any hormonal contraception. Nine out of 10 participants had biological children, and four of the 10 females had been diagnosed with female factor infertility. Mean age of the females was 34.9( ± 4.75 s.d., range 28–41) years. All 10 females donated follicular fluid samples, which were collected during the transvaginal oocyte retrieval that was performed after the follicle maturation protocol (see Jokiniemi et al. 2020a, for the detailed description of the protocol). The mean number of collected mature oocytes was 8.6( ± 3.89 s.d.) per female. After the collection, follicular fluid samples were centrifuged at 500 × *g* for 10 min, and the supernatant was aliquoted and stored in liquid nitrogen (−196 °C) for later use.

### Female rating of male body odours

The above-mentioned 10 females evaluated the pleasantness and intensity of all 11 males’ odours. Females were advised to evaluate male odours as ‘potential reproductive partners’. Rating sessions took place between December 2020 and March 2021. Prior to the ratings, each female received odourless personal hygiene products (LV®) and Clearblue® digital ovulation tests (Swiss Precision Diagnostics GmbH). Twenty-four hours before their estimated ovulation time, the participants were asked to cease using any perfumed products and avoid eating aromatic food (Supplementary Methods 1). Females rated the male odours within 48 h of their positive ovulation test result (i.e. luteinizing hormone surge that occurs ca. 24 h prior to the actual ovulation).

All the cotton pads from both male armpits were cut into two similarly sized pieces (hereafter ‘pad piece’), which were individually placed at the bottom of clean and odourless 50 ml Erlenmeyer flasks, covered with foil and closed with a foil cap (Supplementary Fig. 1). Each pad piece was used in maximum of four rating sessions before being replaced with a new one (all the 11 pads were always changed at the same time). Between rating sessions, pad pieces were stored in their Erlenmeyer flasks at −80 °C. Odour rating was carried out in an odourless room at the North Karelia Central Hospital. Odour samples were allowed to settle down to room temperature for one hour prior to the ratings. Just before the rating session, the room was aired out, and written and oral instructions of the odour rating procedure were given to the raters. Females were asked to rate male odours based on their intensity and pleasantness, using a continuous scale (from ‘non-intensive’ to ‘very intensive’ and ‘non-pleasant’ to ‘very pleasant’) in 10 cm-long line segments printed on papers that were given to the raters. Raters were asked to mark their scores on the line, from which we later measured their scores with 1 mm accuracy (0–100 mm). If the rater could not smell the sample, the answer sheet was left empty. All the females were alone in the room during the rating sessions, all of which lasted a maximum of 20 min. All the participants managed to perform their ratings within this time frame. Females rated all the odour samples a total of four times, with 10 min breaks between the rating rounds. During the breaks, the room was aired out, and the order of the samples was randomised. In other words, participants always rated the samples blind, without knowing the identity (male ID code) of the samples. Additionally, clearly marked odourless cotton pad was always present along with anonymous male samples to control for the pads’ own odour.

### Collection of semen samples and sperm treatment with follicular fluid

All the males provided semen samples after 2–4 days of sexual abstinence by masturbating into clean plastic cups. Semen samples were allowed to liquefy for 45 min at +37 °C, and samples were then washed with two-layer (PureSperm® 40 and 80) density gradient centrifugation according to the manufacturer’s protocol (Nidacon International AB, Mölndal, Sweden). Finally, the sperm pellets (including both motile and non-motile cells) were resuspended in PureSperm® Wash solution to the final concentration of ca. 75 million sperm/ml. Follicular fluid samples (one aliquot/female) were defrosted ca. 15 min prior to the initiation of the follicular fluid treatments. Each of the 10 follicular fluid samples was divided into two independent sub-samples, A and B (20 samples in total). Washed sperm aliquots from each male were then combined 1:1 (vol:vol, 25 µl sperm and 25 µl follicular fluid) with these 20 follicular fluid samples in all possible male-female combinations (full-factorial design: *n* = 11 males × 10 females × two sub-samples = 220 combinations in total). All samples were kept in 1.5 ml Eppendorf tubes at +37 °C during the whole experimental period.

### Sperm motility measurements

The effect of the follicular fluid identity on sperm motility was determined using Computer-Assisted Sperm Analysis (CASA; Integrated Semen Analysis System, ISAS v. 1.2 Proiser, Valencia, Spain), with a negative phase contrast microscope (100× magnification) and a capture rate of 100 frames ^−1^. Threshold to eliminate drifting non-motile cells was set to 10 µm/s (based on straight line velocity, VSL), and the area of examined cells was 7–30 µm^2^. Sperm motility was measured by adding one microlitre of each of the sperm-follicular fluid dilutions to the pre-warmed (+37 °C) Leja 4-chamber (chamber height 20 μm) microscope slides (Leja, Nieuw-Vennep, the Netherlands). We then recorded sperm motility (1 s per recording) at three different time points (60, 180, and 300 min) after the beginning of the follicular fluid treatment. All the replicate recordings were always performed using fresh sperm-follicular fluid dilution aliquots taken from the Eppendorf tubes. These time points were selected based on the earlier observations that the capacitation of human spermatozoa occurs in 40–130 min and lasts up to 240 min (Cohen-Dayag et al., 1995). We took two independent recordings from both sub-samples, resulting in four replicate measurements in total. To avoid a potential time effect on the measured sperm traits, sperm motility in the first (A) sub-samples was always measured in the following order: FF1, FF2,…, FF10, whereas the second (B) sub-samples were measured in the opposite order: FF10, FF9,…, FF1. The proportion of hyperactivated sperm cells in each of the above-mentioned time points was determined based on three motility parameters that have been shown to characterise hyperactivated sperm motility (Kay and Robertson 1998): sperm curvilinear velocity (VCL > 150 µm/s), linearity (LIN < 50%) and amplitude of lateral head displacement (ALH > 2.0). All sperm motility parameters were measured on average from 1876 ± 103 (s.d.) sperm cells per male-female combination, and all the motility measurements were performed on the day of semen collection (i.e. using fresh sperm).

### Genotyping of the study subjects

DNA from all the participants was extracted from EDTA blood samples using PureLink® Genomic DNA Kit (Invitrogen) according to the manufacturer’s protocol. DNA samples were genotyped with the Illumina Global Screening Array-24 (GSA) v2.0 or v3.0 platform at the Institute for Molecular Medicine Finland (FIMM). SNP positions from the data set genotyped with the GSA v2.0 platform were lifted over to the human reference genome build GRCh38/hg38 using the UCSC hgLiftOver tool (https://genome.ucsc.edu/cgi-bin/hgLiftOver). The lifted genotype data were thereafter merged with the rest of the samples genotyped with the GSA v3.0 platform. SNPs not shared between the two data sets were discarded.

HLA alleles from classical (class 1 a: A, B, C; class 2: DRB1, DQA1, DQB1, and DRB1) and non-classical loci (class 1b: E, F, and G) were imputed at four-digit (i.e. protein-level) resolution using a Finnish reference panel (Ritari et al. 2020) and the R package HIBAG v1.26.0 (Zheng et al. 2014). All imputed HLA alleles, except HLA-G UTR alleles, correspond to nonsynonymous variants. Although HLA class 1b alleles were discovered long after the classical HLA genes, recent studies have demonstrated that non-classical HLAs can play a particularly important role in reproduction and fertility (e.g. Nilsson and Hviid 2022). Thus, both classical and non-classical alleles (20 alleles in total) were included in the final analyses, which allowed us to test the overall impact of these 10 genes on HLA-associated mate choice. However, since earlier MHC-based mate choice studies have mainly concentrated on classical MHC genes, we calculated HLA allelic similarities between all 110 male–female combinations separately for total and classical genes by counting the number of alleles shared between each combination. In this calculation, homozygotes were accounted for by counting each allele only once. Pairwise kinship between male-female combinations was estimated using the KING robust kinship estimate (Manichaikul et al. 2010) implemented in plink v2.00a3LM (Chang et al. 2015; Purcell and Chang 2021) using the command ‘make-king-table’. Prior to kinship calculations, the genotype data were quality filtered using plink v1.9 commands ‘hwe 1e-6’ to exclude variants not in Hardy–Weinberg equilibrium and ‘maf 0.05’ to exclude variants with minor allele frequency below 5%. Final kinship calculations included on average 284,103 ± 2033 (s.d.) SNPs per male-female combination.

### Statistical analyses

The effects of male, female, and male-female interaction (random effects) on perceived odour pleasantness and intensity (response variables, Supplementary Methods 2, model #1) were tested in linear mixed models (LMM), with Restricted Maximum Likelihood method. To control for the potential effect of handling and storage of odour samples, as well as the variation between rating rounds, models also included the number of pad thawing times, pad pieces, and rating round as fixed effects. Additionally, to control for the potential effect of female infertility on our results, we also included female factor infertility diagnosis (1 = infertility diagnosis, 0 = no diagnosis) as an additional fixed effect in the models. To test the association between odour pleasantness and genetic parameters (total and classical HLA allelic similarity or pairwise kinship) between male–female combinations, these genetic parameters were included as covariates (one parameter at a time) in the above-mentioned models (Supplementary Methods 2, model #2). In order to test whether the slope between the odour pleasantness and HLA similarity was consistent across different males, we also included total HLA-similarity|male or classical HLA-similarity|male as additional random effects in model #2.

To clarify the potential association between odour intensity and odour pleasantness ratings, we first performed explorative Spearman correlation analysis across all 110 male–female combinations and found that these two odour metrics were negatively correlated (*r* = 0.51, *P* < 0.001; Supplementary Fig. 2). Then the potential effect of odour intensity on perceived odour pleasantness as well as the association between odour pleasantness or intensity and HLA allelic similarity were investigated in more detail in separate models. In these models, HLA-similarity (total and classical) acted as a response variable, and odour intensity, odour pleasantness and their interaction were modelled as covariates (Supplementary Methods 2, model #3). The models also included the same categorical fixed effects and random effects, as described above. The final models were simplified by removing the statistically non-significant odour intensity × odour pleasantness interaction. The association between HLA similarity and overall genetic relatedness was tested in the models, where total or classical HLA similarity acted as response variables and pairwise kinship as a fixed covariate (Supplementary Methods 2, model #4). Model also included random effects of males and females.

Prior to the sperm motility models, we performed principal component analyses (with varimax rotation) for the following sperm parameters: curvilinear velocity (VCL), straight line velocity (VSL), average path velocity (VAP), amplitude of lateral head displacement (ALH) and the proportion of hyperactivated sperm cells. Principal component analyses resulted in one principal component (‘sperm motility component’, eigenvalue = 4.37), which explained 87.3% of the total variation in the above-mentioned sperm traits and which was positively affected by all five parameters (component loadings: VSL = 0.775; hyperactivation = 0.926; ALH = 0.979; VAP = 0.984; VCL = 0.990).

The full model for sperm motility component (response variable, Supplementary Methods 2, model #5) included random effects of male, female, and male-female interaction, as well as timepoint, sub-sample (A or B), and female infertility diagnosis, which were modelled as fixed effects. Timepoint-specific models for sperm motility were otherwise similar but included only sub-sample and female infertility diagnosis as fixed effects (Supplementary Methods 2, models #6–9). Furthermore, the association between VCL and the above-mentioned genetic parameters was tested by including genetic parameters as covariates to the timepoint-specific models.

Finally, the model testing for the association between timepoint-specific sperm motility (response variable) and odour pleasantness included sub-sample and female infertility diagnosis as fixed effects and odour pleasantness as a covariate (Supplementary Methods 2, model #8). To test whether the slope between sperm motility and odour pleasantness was similar across different males, we also modelled the interaction between odour pleasantness and male (odour pleasantness|male) as an additional random effect. Similarly, to investigate whether the association between sperm motility and odour pleasantness is affected by odour intensity, we added odour pleasantness-odour intensity interaction term in the above-mentioned models (Supplementary Methods 2, model #9).

All statistical analyses were performed in RStudio (R version 4.2.2, R Core Team 2018), using the lmerTest (v. 3.1.1) package (with type III ANOVA) and marginal and conditional *R*^2^-values of the models were calculated with MuMin package (v. 1.43.17) (Barton 2009; Kuznetsova et al. 2017). Conditional *R*^2^-values were used in model selection to determine whether to include female infertility diagnosis in our final models (see above). Graphs were visualised using ggplot2 (v. 3.3.2) package (Wickham 2016). All *P* values are from two-tailed tests, with *α* = 0.05. Model assumptions were graphically verified using Q-Q plots and residual plots. To ensure that the predictor variables of our HLA-similarity models (Supplementary Methods 2, model #3) were not correlated (multicollinearity), we performed generalised VIF (GVIF) and adjusted generalised standard error inflation factor (aGSIF) diagnostics for these models using the car package (v. 3.1–3). Finally, to ensure the generalisability of all the above-mentioned fixed effects, we performed jackknife removal of each male and female for all of our models and visually compared the jackknife estimates of fixed effects with the full model.

## Results

### Female odour ratings

**Fig. 1:**
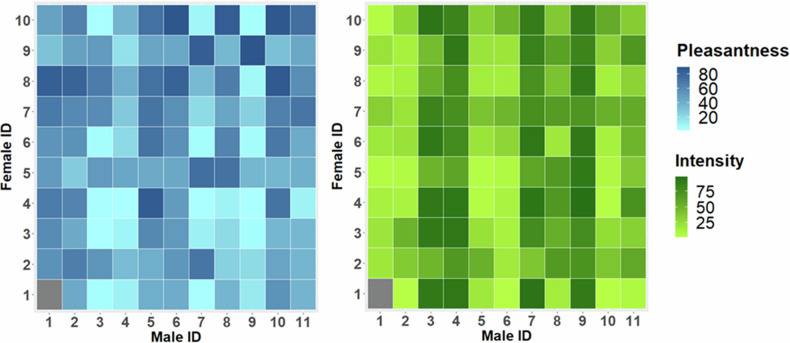
Perceived odour pleasantness (left) and intensity (right) in all (*n* = 110) male–female combinations. Light hues (pale blue and green) indicate unpleasant and less intense odours, respectively. Similarly, darker hues are associated with pleasant and more intense odours. Female 1 failed to rank the odour sample of Male 1 in all four rating rounds (indicated as grey colour).

Both odour pleasantness and intensity were affected by male and male–female interaction, but not by female effect (Table 1 and Fig. 1). Additionally, odour pleasantness was not affected by female infertility diagnosis, rating round, pad piece or number of thawing times. Odour intensity was significantly affected by pad piece, but not by female diagnosis, rating round or number of thawing times. Both odour ratings also showed high degree of consistency across four rating rounds (intra-class correlation coefficient, pleasantness: 0.87, *P* < 0.001; intensity: 0.92, *P* < 0.001). Together, these results show that female odour ratings did not differ across the four rating rounds and thawing of the pads did not affect female odour perception. Odour pleasantness ratings were also unaffected by the different pad halves used in the different rating sessions.

**Table 1:** Linear mixed model statistics for the effects of male, female, and male–female interaction (random effects) on male odour pleasantness and intensity.

Effects	Pleasantness				Intensity			
Random	*χ*^*2*^	d.f.	*P* value	% var	*χ*^*2*^	d.f.	*P* value	% var
Male	18.68	1	**<0**.**001**	18.25	103.5	1	**<0**.**001**	58.65
Female	1.32	1	0.25	5.24	0.00	1	1.0	0.00
M × F	104.8	1	**<0**.**001**	33.58	53.98	1	**<0**.**001**	13.48
Residual				34.96				24.67
**Fixed**	*F*	d.f.	*P* value	% var	*F*	d.f.	*P* value	% var
Diagnosis	0.82	1, 3	0.43		0.56	1, 92	0.45	
R. round	1.08	3, 310	0.36		0.27	3, 310	0.84	
Pad piece	0.23	2, 3	0.81		5.33	2, 92	**0.006**	
T. times	2.46	3, 3	0.24		2.33	3, 92	0.080	
Total				7.96				3.20

Model also included fixed effects of female diagnosis, rating round (R. round), pad piece, and number of pad thawing times (T. times). Statistically significant *P*-values are indicated with bold font. %var: proportion of variance explained by random effects and total variance explained by four fixed effects.

**Fig. 2:**
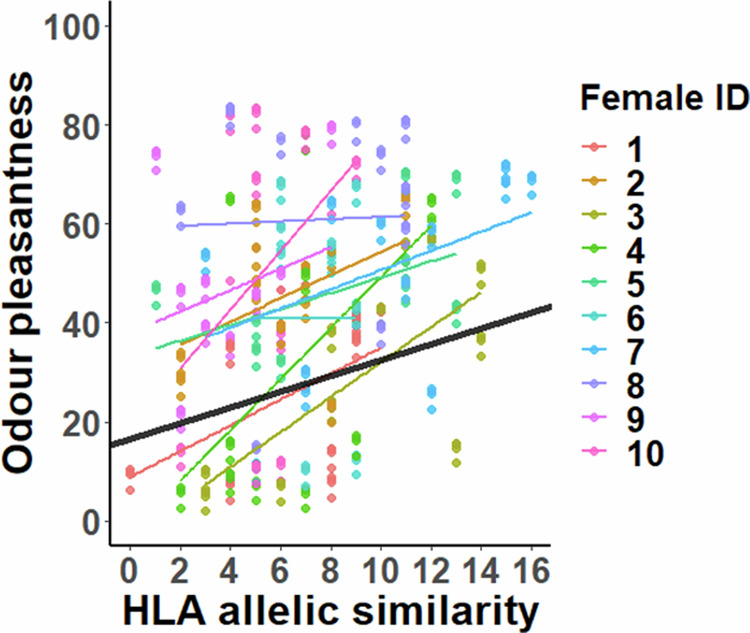
The association between odour pleasantness and male–female total HLA allelic similarity between males and females. Female-specific associations are identified by different colours, and the black line shows the average slope across all females. The slope of the association did not differ between females (*χ*^*2*^ = 0.45, *P* = 0.80). Datapoints represent fitted values obtained from the LMM.

We found a statistically significant positive association between odour pleasantness and total HLA allelic similarity (*t*_100.7_ = 2.20, *P* = 0.029, Fig. 2). A similar association was also found for classical HLA alleles, but the slope did not reach statistical significance (*t*_98.4_ = 1.92, *P* = 0.058). Furthermore, the slope between the HLA similarity and odour pleasantness (HLA-similarity|male) was consistent across males (all HLA alleles: *χ*^2^_2_ = 0.12, *P* = 0.94; classical HLA alleles: *χ*^2^_2_ < 0.01, *P* = 0.99), demonstrating that females preferred HLA similar odours also within each male. Collectively, these results indicate that females rated the body odours of HLA-similar males as more pleasant than the odours of HLA-dissimilar males and that odour intensity differences across males did not affect female odour pleasantness ratings. Odour pleasantness was not associated with the pairwise kinship estimate of the male–female combinations (*t*_61.0_ = 0.40, *P* = 0.69). However, we found that the kinship estimate was positively associated with both total (*t*_402.4_ = 6.69, *P* < 0.001) and classical HLA similarity (*t*_422.2_ = 5.74, *P* < 0.001), indicating that MHC-similar males and females tend to be genetically more closely related than more dissimilar individuals.

Finally, the interaction effect between odour pleasantness and intensity on HLA similarity was statistically non-significant (all HLA alleles: *t*_398.5_ = 1.26, *P* = 0.21; classical HLA alleles: *t*_397.4_ = 1.68, *P* = 0.094). In the final models (without odour pleasantness-intensity interaction), odour intensity was not associated with total HLA similarity (*t*_357.0_ = −0.97, *P* = 0.33), but the positive association between odour pleasantness and HLA similarity was still present (*t*_397.7_ = 2.33, *P* = 0.020). For classical HLA alleles, neither odour pleasantness nor odour intensity was associated with HLA similarity (pleasantness: *t*_396.8_ = 1.71, *P* = 0.088; intensity: *t*_386.6_ = −1.74, *P* = 0.082). Together above-mentioned results indicate that odour intensity did not affect the perceived odour attractiveness and that HLA similarity was not associated with odour intensity. In other words, a demonstrated positive association between odour pleasantness and HLA allelic similarity was found to be independent of odour intensity.

### Sperm motility

Sperm motility was negatively affected by the timepoint (*F*_2,1208_ = 1842.5, *P* < 0.001). Timepoint-specific models showed that motility was affected by male at all timepoints, whereas male–female interaction was significant at the last two timepoints and female effect at 60 min and at 300 min timepoints (Table 2).

**Table 2:** Linear mixed model statistics for the effects of male, female, and male-female interaction (M × F) (random effects) on sperm motility at three different timepoints (60, 180, and 300 min) after the sperm treatments with follicular fluid.

Effects	Motility component 60 min				Motility component 180 min				Motility component 300 min			
Random	*χ*^*2*^	d.f.	*P* value	% var	*χ*^*2*^	d.f.	*P-*value	% var	*χ*^*2*^	d.f.	*P* value	% var
Male	202.66	1	**<0.001**	74.64	70.67	1	**<0**.**001**	33.03	48.30	1	**<0**.**001**	26.70
Female	11.78	1	**<0.001**	2.09	1.06	1	0.30	1.16	4.96	1	**0.026**	3.94
M×F	3.74	1	0.053	1.88	4.77	1	**0**.**029**	5.87	32.09	1	**<0**.**001**	14.61
Residual				20.17				54.96				41.06
**Fixed**	*t*	d.f.	*P* value		*t*	d.f.	*P* value		*t*	d.f.	*P* value	
Intercept	4.21	11.2	**0**.**001**		3.30	12.5	**0.006**		−5.00	15.1	**<0.001**	
Sub-sample	4.87	329	**<0**.**001**		−6.26	329	**<0.001**		−11.23	329	**<0.001**	
Diagnosis	−0.71	8.0	0.50		0.49	8.0	0.64		1.75	8.0	0.12	
Total				1.23				4.98				13.70

Statistically significant *P* values are indicated with bold font. %var: proportion of variance explained by random effects and fixed effects (sub-sample and female infertility diagnosis).

**Fig. 3:**
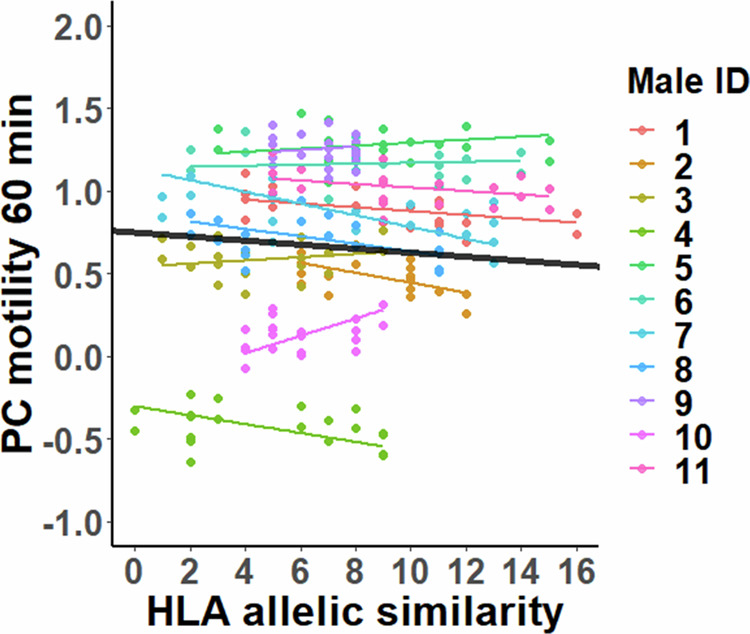
The association between sperm motility 60 min after the initiation of the follicular fluid treatment and male–female total HLA allelic similarity. Male-specific associations are identified by different colours, and the black line shows the average slope across all males. The slope of the association did not differ between males (*χ*^*2*^ = 2.68, *P* = 0.26). Datapoints represent fitted values obtained from the LMM.

Sperm motility was negatively associated with total male-female HLA allelic similarity 60 min after the initiation of sperm follicular fluid treatment (*t*_97.5_ = −2.42, *P* = 0.018, Fig. 3). No statistically significant associations were found at the other two timepoints (180 min: *t*_95.7_ = 0.19, *P* = 0.85; 300 min: *t*_102.7_ = −0.28, *P* = 0.78). Sperm motility was not associated with classical HLA allelic similarity, although similar negative slope was detected in 60 min (60 min: *t*_97.9_ = −1.78, *P* = 0.078; 180 min: *t*_92.3_ = 0.30, *P* = 0.77; 300 min: *t*_103.3_ = −0.39, *P* = 0.70). Furthermore, no associations were found between sperm motility and pairwise kinship of the male–female combinations at any of the timepoints (60 min: *t*_88.2_ = −0.51, *P* = 0.61; 180 min: *t*_44.6_ = 0.88, *P* = 0.39, 300 min: *t*_72.7_ = 0.78, *P* = 0.44).

### Interaction between pre- and post-mating mate choice

**Fig. 4:**
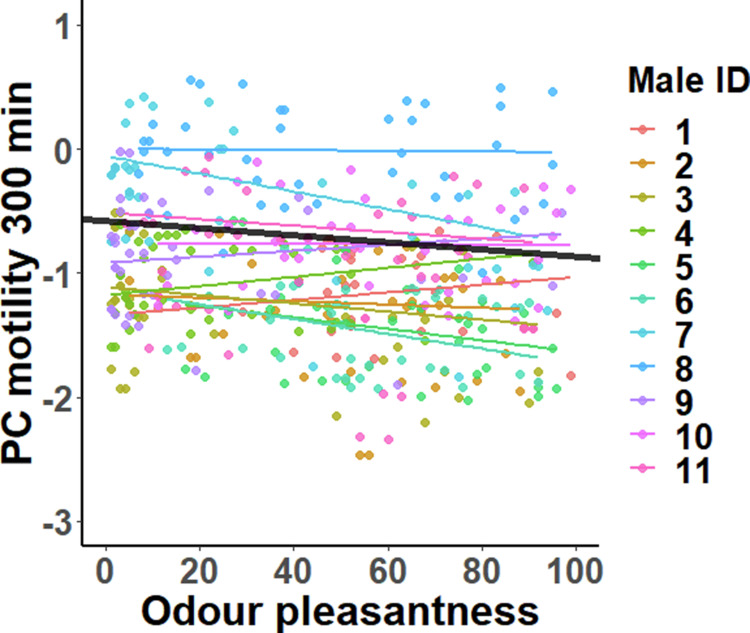
The association between perceived male odour pleasantness and sperm motility 300 min after the initiation of the follicular fluid treatment. Male-specific associations are identified by different colours, and the black line shows the average slope across all males. Datapoints represent fitted values obtained from the LMM. The slope of the association did not differ between males (*χ*^*2*^ = 4.57, *P* = 0.10).

Male body odour pleasantness was negatively associated with sperm motility 300 min after the follicular fluid treatment (*t*_365.0_ = −2.05, *P* = 0.041, Fig. 4), but no associations were found at the other two timepoints (60 min: *t*_279.4_ = −1.45, *P* = 0.15 and 180 min: *t*_265.4_ = −0.63, *P* = 0.53). The slope of the association did not differ between males at any of the three timepoints (60 min: *χ*^*2*^_2_ = 0.90, *P* = 0.64; 180 min: *χ*^*2*^_2_ = 0.22, *P* = 0.90; 300 min: *χ*^*2*^_2_ = 4.52*, P* = 0.10), indicating that the association between odour pleasantness and sperm motility was consistent across males. Finally, the interaction effect between odour pleasantness and intensity on sperm motility was statistically non-significant in all timepoints (60 min: *t*_354.4_ = −0.85, *P* = 0.40; 180 min: *t*_224.9_ = 1.26, *P* = 0.21; 300 min: *t*_373.9_ = 0.63, *P* = 0.53), indicating that the association between odour attractiveness and sperm motility was not affected by male odour intensity.

## Discussion

Our results show that females perceived the body odours of HLA-similar males as more pleasant than the odours of more dissimilar males. In contrast (and in line with the findings by Jokiniemi et al. 2020a), sperm motility at 60 min after the follicular fluid treatment was higher in HLA-dissimilar male-female combinations. Mate choice both at the pre-mating and post-mating level was found to be stronger when both classical and non-classical HLA alleles were included in the analyses, indicating that HLA-associated mate choice may not be limited to widely studied classical HLA alleles. Finally, our results show that perceived odour pleasantness was negatively associated with sperm motility at the end of the sperm follicular fluid treatment. Taken together, these results demonstrate that HLA-mediated pre-mating and post-mating mate choice may act in opposing directions, indicating that females may base their mate choice on different criteria during different episodes of sexual selection (Mahdjoub et al. 2023).

Odour-based mating preferences are often assumed to be disassortative, as genetic dissimilarity between mating partners is expected to increase offspring health and/or survival via heterozygosity advantage (Allen et al. 2019). However, evidence for MHC-based disassortative odour preferences is contradictory (Winternitz et al. 2017; Allen et al. 2019), and in some animal taxa, preferences for MHC-similar partners have also been reported (Bonneaud et al. 2006; Sin et al. 2015; Leclaire et al. 2017). In humans, it has been suggested that odour preferences towards HLA-similar partners may be an artefact resulting from ethnic heterogeneity of the study subjects (pooling of different ethnic groups together: Winternitz et al. 2017), female usage of hormonal contraceptives during the odour rating (Wedekind et al. 1995), or that it may reflect overall context-dependency of HLA-associated mate choice (HLA-based non-random mating may operate only under certain socio-cultural contexts: Dandine-Roulland et al. 2019).

In the present study, all the participants were Caucasian (they had Finnish names and Caucasian appearance), and females were not using any hormonal contraception. Females also rated male odours just after their confirmed ovulation and had a regular menstrual cycle. Thus, it is unlikely that ethnic heterogeneity or ovulatory cycle-dependent shifts in female mating preferences (Gildersleeve et al. 2014) could explain observed odour preferences for HLA-similar males. However, the mean age of participating females was relatively high (ca. 35 years), whereas most of the earlier human mate choice studies have been conducted among younger adults (Sprecher et al. 2019). Thus, it is possible that this discrepancy can be at least partly explained by age-dependent changes in female mating preferences. Supporting this view, Sprecher et al. (2019) demonstrated that mate selectivity decreases with age in humans. However, as decreased selectivity cannot necessarily be expected to reverse the direction of mate preferences, further studies investigating how HLA-based mating preferences vary in relation to female age would be required to confirm this possibility. Furthermore, given that in the present study, male odour collection was initiated relatively soon (24 h) after the odourless deodorant usage, future studies should pay more attention to the formation of skin natural microbiota prior to odour collection.

In contrast to the pre-mating mate choice, our results revealed that total HLA dissimilarity between males and females was associated with increased sperm motility in the presence of follicular fluid, which is indicative of high fertilisation success of such male-female combinations. Given that male-female HLA dissimilarity is generally thought to indicate better genetic compatibility of the partners (e.g. Schwensow et al. 2008; Agbali et al. 2010; Strandh et al. 2012; Huang et al. 2021), our results indicate that the genetic incompatibility of the males and females may be reliably revealed only after copulation (Huang et al. 2021). Supporting this conclusion, an earlier study has demonstrated that similar selection against HLA-based genetic incompatibility also occurs in the cervical mucus and thus already in the lower part of the female reproductive tract (Jokiniemi et al. 2020b). In line with these findings, it has been suggested that GMMC may provide a much more accurate way to ‘evaluate’ the reproductive compatibility of the partners than any of the individual-level mate choice processes (Holt and Fazeli 2015; Kekäläinen and Evans 2018).

The above-mentioned reversal in the direction of HLA-mediated mate choice may also indicate that pre- and post-copulatory mate choice episodes act together to ensure that resulting offspring have certain optimal level of HLA heterozygosity (Radwan et al. 2020). Accordingly, although MHC heterozygous individuals have better ability to present antigens to the immune system, they may have smaller T-cell repertoires, due to thymic selection (Penn and Potts 1999; Migalska et al. 2019) and higher risk of various autoimmune disorders (Murray et al. 2020). This may favour the evolution of mate choice targeting intermediate MHC diversity (Aeschlimann et al. 2003; Wegner et al. 2003; Roberts et al. 2005b; Phillips et al., 2017). Such selection has earlier been demonstrated to occur both before (Reusch et al. 2001; Jacob et al. 2002; Forsberg et al. 2007) and after mating (Lenz et al., 2018). Interestingly, Lenz et al. (2018) showed that selection for intermediate MHC diversity can occur exclusively (and most accurately) at the gamete level via haplotype-specific sperm selection that bias fertilisation towards gametes with complementary MHC genes. This indicates that GMMC may have similar function in humans, facilitating non-random fertilisation of offspring that have intermediate HLA diversity. In any case, the overall effect of pre- and post-mating episodes of sexual selection on MHC diversity has remained unclear, and to the best of our knowledge, none of the earlier studies have experimentally investigated both episodes of MHC-based mate choice within the same individuals. Although present results indicate that the direction of the mate choice for MHC genes may reverse between the selection episodes, more studies are needed to better understand the overall impact of pre- and post-mating mate choice processes on MHC diversity. Furthermore, since many of our study subjects were recruited via the local infertility clinic, some caution is advisable when generalising our findings to human populations at large.

Finally, rather than optimising the MHC diversity of the offspring per se, it is possible that MHC-associated mating preferences may also represent a by-product of inbreeding avoidance, preventing mating between close relatives (Huang et al. 2021). Although in the present study, the degree of genetic relatedness (kinship) between male-female combinations was not associated with either odour pleasantness or sperm motility, genetic relatedness of the males and females predicted their HLA similarity. Therefore, it is possible that observed pre- and post-copulatory mate choice for HLA genes may have an additional function in inbreeding avoidance.

In conclusion, our results show that pre- and post-copulatory mate choice episodes can act in opposing directions and that female-induced sperm selection may not necessarily favour the males that were perceived as most attractive in odour-based mate choice trials. In this way, the present findings can help to understand the interaction of pre- and post-copulatory episodes of sexual selection in humans and potentially also in other species. Our findings also indicate that female-mediated post-copulatory sperm selection process may have a definitive role in ‘evaluating’ the compatibility of the paternal and maternal genomes prior to fertilisation. A deeper understanding of these selection mechanisms may have novel implications for a better understanding of infertility.

## Supplementary information

Supplementary information

## Data Availability

The original data of the study is available at figshare: https://figshare.com/s/02f40dd5fd571ba19008.
