# Supplementary material for: Female-mediated selective sperm activation may remodel major histocompatibility complex-based mate choice decisions in humans
Source: Heredity (Edinb). 2025 May 9;134(6):321–30. doi: 10.1038/s41437-025-00759-9 (PMC12137739; doi:10.1038/s41437-025-00759-9)
Supplement: Supplementary file 1 — Supplementary information [file 41437_2025_759_MOESM1_ESM.docx]

**Supplementary Methods 1**

**List of products given to the study subjects and restrictions**

**A. Males**

**Products given to the participants:**

- washing detergent for clothes (LV, Berner®)
- odourless shower gel (LV® Berner)
- odourless body lotion (LV® Berner)
- odourless face lotion (LV® Berner)
- odourless shampoo (LV® Berner)
- odourless conditioner (LV® Berner)
- odourless face wash (LV® Berner)
- odourless deodorant (LV® Berner)
- pre-washed bedding
- pre-washed cotton t-shirt
- cotton pads for odour collection
- skin tape to attach cotton pads (Leukoplast skin sensitive REF 76173-00)
- foil to enclose the odour samples (cotton pads)
- zip bags for the storage of the cotton pads and t-shirt
- sterile container for a semen sample

**Restrictions 72 h before the odour sample collection**

Stop using antiperspirants. We provide you with an odourless deodorant you can use until 24 h prior to the odour sample collection.

**Restrictions 24 h before the sample collection and during the sample collection**

Only use the personal hygiene products we provide. Moreover, stop using the deodorant and all the other products in your armpits that could affect the formation of sweat.

Avoid using the following scented products:

- shower gels
- deodorants / antiperspirants
- perfumes, aftershaves
- shaving foams
- skin lotions
- hair products (such as hair spray, shampoos, and conditioners)
- other scented skin products

Avoid the following food items and intoxicants:

- garlic and onion
- cabbage and asparagus
- strong spices, such as pepper, chili, curry, vinegar, and herbs
- salami, marinated fish, and marinated meat
- blue cheese and Brie cheese
- alcohol, tobacco, and snuff
- pharmaceuticals (if possible)

**B. Females**

**Products given to the participants:**

- washing detergent for clothes (LV®, Berner)
- odourless shower gel (LV®, Berner)
- odourless body lotion (LV®, Berner)
- odourless face lotion (LV®, Berner)
- odourless shampoo (LV®, Berner)
- odourless conditioner (LV®, Berner)
- odourless face wash (LV®, Berner)
- odourless antiperspirant (LV®, Berner)
- Clearblue® Digital Ovulation test package (SPD Swiss Precision Diagnostics GmbH)
- plastic cups to collect urine for ovulation tests

**Restrictions 24 h before estimated ovulation and on the day of odour rating**

Stop using all the scented products, such as body sprays, body lotions, perfumes, shampoos, and shower gels and avoid wearing clothes that have been washed with scented detergent. Only use the personal hygiene products provided for you.

Avoid using the following scented products:

- shower gels
- deodorants / antiperspirants
- perfumes
- shaving foams
- shampoos and conditioners
- skin lotions
- hair products (such as hair spray)
- other scented skin products

Avoid the following food items and intoxicants:

- garlic and onion
- cabbage and asparagus
- strong spices, such as pepper, chili, curry, vinegar, and herbs
- salami, marinated fish, and marinated meat
- blue cheese and Brie cheese
- alcohol, tobacco, snuff
- pharmaceuticals (if possible)

**On the day of the odour rating session**

- Avoid using scented products; wash your hair and body only with odourless products and use odourless deodorant
- Wear odourless clothes
- Avoid alcohol, tobacco, and snuff
- Do not eat anything for one hour before the rating session
- Avoid caffeinated products (coffee, energy drinks, tea, etc.) for one hour before the rating session
- Avoid strong external odour contaminations (i.e. smoke, paints, glues, and solvents)
- Avoid cooking and eating aromatic food

**Supplementary Figure 1**

**
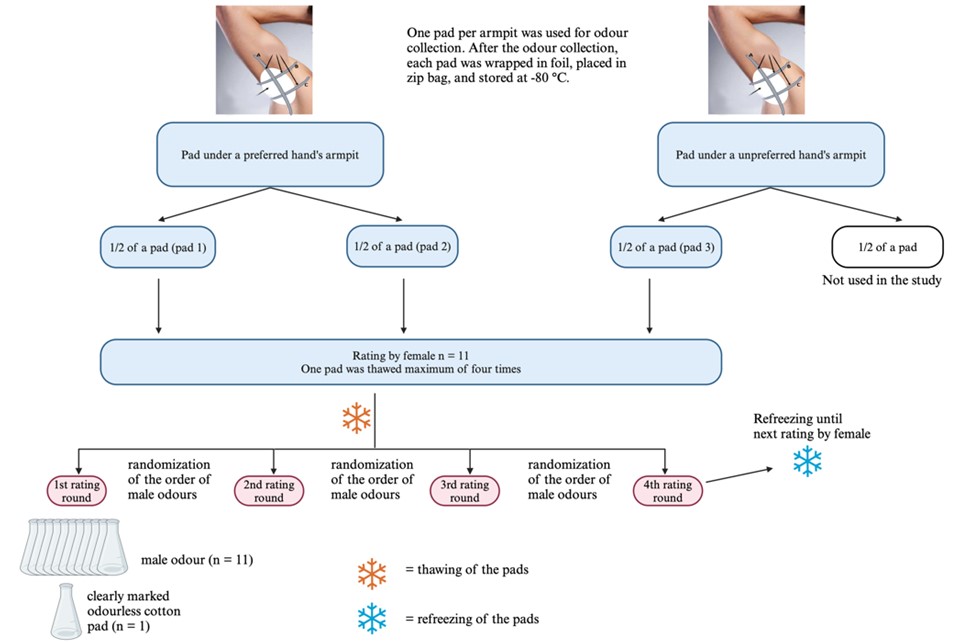
**

**Supplementary Figure 1. A flow chart of the experiment. Male odour samples were collected with cotton pads from both armpits (‘preferred and unpreferred hand’ = dominant and non-dominant hand), which were wrapped in foil, placed in zip bags, and stored at -80 °C. Both pads were cut into two similarly sized pieces (four pad pieces in total). Each piece was used in maximum of four rating sessions (stored at -80 °C between the sessions) before they were replaced with a new one (all the pads were always changed at the same time). Females rated male odours in total of four times (1^st^ – 4^th^ rating rounds). Order of odour samples were randomized (mixed) between rating rounds. In addition to male pads, a clearly marked, odourless ‘control’ pad was also present in all the rating sessions as a reference (to control for the pads own odour). An orange snowflake indicates the pad thawing and a blue snowflake indicates pad freezing.**

**Supplementary Figure 2**

**
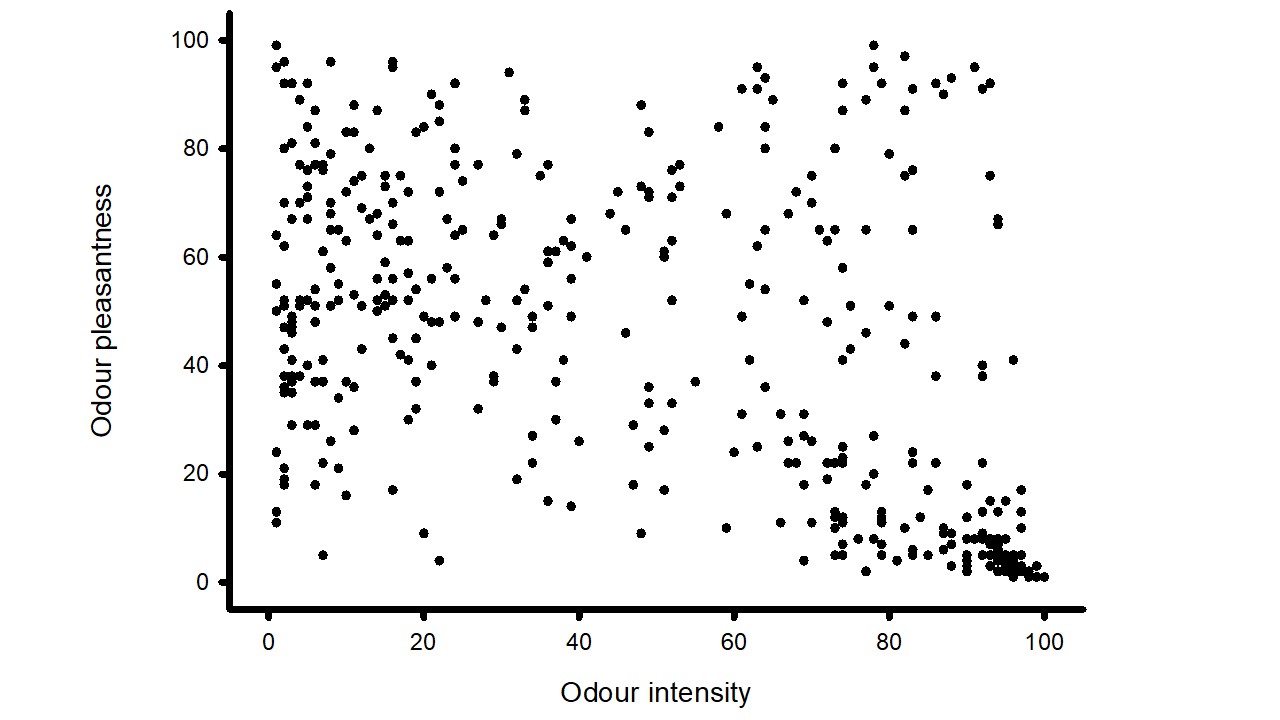
**

**Supplementary Figure 2. The association between odour pleasantness and odour intensity across 110 male-female combinations.**

**Supplementary Methods 2:**

**R scripts for the models used in article “Female-mediated selective sperm activation may remodel major histocompatibility complex -based mate choice decisions in humans”.**

**#1. Models for odour pleasantness or intensity**

Odour_pleasantness ~ female_diagnosis + rating_round + pad_number + thawing_amount + (1 | male) + (1 | female) + (1 | male:female)

Odour_intensity ~ female_diagnosis + rating_round + pad_number + thawing_amount + (1 | male) + (1 | female) + (1 | male:female)

**#2. Models for odour pleasantness (i) or intensity (ii) with HLA allelic similarity as covariate**

1. Odour_pleasantness ~ HLA_similarity + female_diagnosis + rating_round + thawing_amount + pad_number + (1 | male) + (1 | female) + (1 | male:female)
2. Odour_intensity ~ HLA_similarity + female_diagnosis + rating_round + thawing_amount + pad_number + (1 | male) + (1 | female) + (1 | male:female)

**#3. Models to test the association between odour pleasantness and intensity with (i) and without (ii) odour pleasantness × odour intensity interaction**

1. HLA_similarity ~ odour_pleasantness * odour_intensity + rating_round + thawing_amount + pad_number + female_diagnosis + (1|male) + (1|female)
2. HLA_similarity ~ odour_pleasantness + odour_intensity + rating_round + thawing_amount + pad_number + female_diagnosis + (1|male) + (1|female)

**#4. Model testing for the association between genetic relatedness (kinship) and HLA similarity**

HLA similarity ~ kinship + (1 | male) + (1 | female)

**#5. Full model for sperm motility (principal component, PC)**

PC_motility ~ sub_sample + timepoint + female_diagnosis + (1 | male) + (1 | female) + (1 | male:female)

**#6. Timepoint-specific models for sperm motility (principal component, PC)**

PC_motility_60 ~ sub_sample + female_diagnosis + (1 | male) + (1 | female) + (1 | male:female)

PC_motility_180 ~ sub_sample + female_diagnosis + (1 | male) + (1 | female) + (1 | male:female)

PC_motility_300 ~ sub_sample + female_diagnosis + (1 | male) + (1 | female) + (1 | male:female)

**#7. Timepoint-specific models for sperm motility (principal component, PC) with HLA similarity (A) or Kinship (B) as covariate**

1. **HLA similarity**

PC_motility_60 ~ sub_sample + HLA_similarity + female_diagnosis + (1 | male) + (1 | female) + (1 | male:female)

PC_motility_180 ~ sub_sample + HLA_similarity + female_diagnosis + (1 | male) + (1 | female) + (1 | male:female)

PC_motility_300 ~ sub_sample + HLA_similarity + female_diagnosis + (1 | male) + (1 | female) + (1 | male:female)

1. **Kinship**

PC_motility_60 ~ sub_sample + kinship + female_diagnosis + (1 | male) + (1 | female) + (1 | male:female)

PC_motility_180 ~ sub_sample + kinship + female_diagnosis + (1 | male) + (1 | female) + (1 | male:female)

PC_motility_300 ~ sub_sample + kinship + female_diagnosis + (1 | male) + (1 | female) + (1 | male:female)

**#8. Interaction between pre- and post-mating mate choice (timepoint-specific)**

PC_motility_60 ~ sub_sample + odour_pleasantness + female_diagnosis + (1 | male) + (1 | female) + (1 | male:female)

PC_motility_180 ~ sub_sample + odour_pleasantness + female_diagnosis + (1 | male) + (1 | female) + (1 | male:female)

PC_motility_300 ~ sub_sample + odour_pleasantness + female_diagnosis + (1 | male) + (1 | female) + (1 | male:female)

**#9.** **Models to test the effect of odour pleasantness - odour intensity interaction on sperm motility**

1. PC_motility_60 ~ sub_sample + odour_pleasantness * odour_intensity + female_diagnosis + (1 | male) + (1 | female) + (1 | male:female)

PC_motility_180 ~ sub_sample + odour_pleasantness * odour_intensity + female_diagnosis + (1 | male) + (1 | female) + (1 | male:female)

PC_motility_300 ~ sub_sample + odour_pleasantness * odour_intensity + female_diagnosis + (1 | male) + (1 | female) + (1 | male:female)

1. PC_motility_60 ~ sub_sample + odour_pleasantness + odour_intensity + female_diagnosis + (1 | male) + (1 | female) + (1 | male:female)

PC_motility_180 ~ sub_sample + odour_pleasantness + odour_intensity + female_diagnosis + (1 | male) + (1 | female) + (1 | male:female)

PC_motility_300 ~ sub_sample + odour_pleasantness + odour_intensity + female_diagnosis + (1 | male) + (1 | female) + (1 | male:female)
